# Aquaporin 5 Interacts with Fluoride and Possibly Protects against Caries

**DOI:** 10.1371/journal.pone.0143068

**Published:** 2015-12-02

**Authors:** Ida Anjomshoaa, Jessica Briseño-Ruiz, Kathleen Deeley, Fernardo A. Poletta, Juan C. Mereb, Aline L. Leite, Priscila A. T. M. Barreta, Thelma L. Silva, Piper Dizak, Timothy Ruff, Asli Patir, Mine Koruyucu, Zerrin Abbasoğlu, Priscila L. Casado, Andrew Brown, Samer H. Zaky, Merve Bayram, Erika C. Küchler, Margaret E. Cooper, Kai Liu, Mary L. Marazita, İlknur Tanboğa, José M. Granjeiro, Figen Seymen, Eduardo E. Castilla, Iêda M. Orioli, Charles Sfeir, Hongjiao Owyang, Marília A. R. Buzalaf, Alexandre R. Vieira

**Affiliations:** 1 Department of Oral Biology, School of Dental Medicine, University of Pittsburgh, Pittsburgh, PA, United States of America; 2 ECLAMC (Latin American Collaborative Study of Congenital Malformations) at CEMIC (Center for Medical Education and Clinical Research), Buenos Aires, Argentina; 3 ECLAMC at Hospital de Area El Bolson, El Bolson, RN, Argentina; 4 Bauru School of Dentistry, University of São Paulo, Bauru, SP, Brazil; 5 Department of Pedodontics, Medipol Istanbul University, Istanbul, Turkey; 6 Department of Pedodontics, Istanbul University, Istanbul, Turkey; 7 Department of Pediatric Dentistry, Yeditepe University, Faculty of Dentistry, Istanbul, Turkey; 8 Dental School, Clinical Research Unit, Federal Fluminense University - UFF, Niterói, RJ, Brazil; 9 Biology Institute, Federal Fluminense University - UFF, Niterói, RJ, Brazil; 10 Department of Endodontics, School of Dental Medicine, University of Pittsburgh, Pittsburgh, PA, United States of America; 11 Department of Pediatric Dentistry, Marmara University, Faculty of Dentistry, Istanbul, Turkey; 12 National Institute of Metrology (INMETRO), Niterói, RJ, Brazil; 13 ECLAMC at INAGEMP-CNPq (National Institute of Population Medical Genetics) at Department of Genetics, Oswaldo Cruz Foundation, Rio de Janeiro, Brazil; 14 ECLAMC at INAGEMP-CNPq (National Institute of Population Medical Genetics) at Department of Genetics, Institute of Biology, Center of Health Sciences, Federal University of Rio de Janeiro, Rio de Janeiro, RJ, Brazil; UNC School of Dentistry, University of North Carolina-Chapel Hill, UNITED STATES

## Abstract

Aquaporins (AQP) are water channel proteins and the genes coding for *AQP2*, *AQP5*, and *AQP6* are clustered in 12q13. Since *AQP5* is expressed in serous acinar cells of salivary glands, we investigated its involvement in caries. DNA samples from 1,383 individuals from six groups were studied. Genotypes of eight single nucleotide polymorphisms covering the aquaporin locus were tested for association with caries experience. Interaction with genes involved in enamel formation was tested. The association between enamel microhardness at baseline, after creation of artificial caries lesion, and after exposure to fluoride and the genetic markers in *AQP5* was tested. Finally, AQP5 expression in human whole saliva, after exposure to fluoride in a mammary gland cell line, which is known to express AQP5, and in *Wistar* rats was also verified. Nominal associations were found between caries experience and markers in the *AQP5* locus. Since these associations suggested that AQP5 may be inhibited by levels of fluoride in the drinking water that cause fluorosis, we showed that fluoride levels above optimal levels change AQP5 expression in humans, cell lines, and rats. We have shown that *AQP5* is involved in the pathogenesis of caries and likely interacts with fluoride.

## Introduction

Aquaporin 5 (AQP5) is a water channel protein expressed in the apical membranes of serous acinar cells in salivary and lacrimal glands, type I alveolar epithelial cells, surface corneal epithelial cells, and during tooth development [1–5]. The genes for AQP2, AQP5, and AQP6 are clustered in a small 27 kilobases region at 12q13 [6]. Although normal in appearance, *Aqp5* null mouse’s saliva is remarkably viscous and of lower volume than saliva collected from wild type and heterozygous mice, however total protein secretion and amylase activity are not affected. The abnormal saliva volume, osmolarity, and electrolyte content in *Aqp*5 null mice implicate the involvement of AQP5 in transcellular fluid secretion across serous-type acinar cells [7]. Serous acinar cells contain multiple salt-transporting proteins for fluid secretion, whereas mucous cells secrete proteins, including amylase [8]. The normal protein, pH, and amylase content of saliva from *Aqp5* null mice is consistent with the absence of AQP5 in mucous cells. Primary saliva should be near isotonic, becoming progressively hypotonic during its passage through the salivary duct. The hypertonic saliva from *Aqp5* null mice suggests that active acinar cell salt secretion into the gland lumen occurs without adequate amounts of water [7]. In humans, the amount of AQP5 in unstimulated saliva is higher during waking hours and decreases during sleeping hours. AQP5 amounts tend to decrease with age, coinciding with the overall decrease in total volume of unstimulated saliva secretion with aging [9]. It has been suggested that AQP5 levels in saliva could be used as an index of the salivary flow rate of humans. AQP5 levels in saliva are decreased in type 1 diabetics and patients with Sjögren’s syndrome, concomitant with a decrease in the salivary secretion of these patients. In Alzheimer patients treated with donepezil, salivary secretion and salivary AQP5 levels are increased compared with those from same-age subjects without Alzheimer’s disease [10].

Saliva has bactericidal/bacteriostatic activities, agglutination and subsequent clearance of bacteria, selective recruitment of bacteria to the enamel pellicle, and resistance to decreased plaque pH [11,12], and functions in protection of the soft and hard oral surfaces from bacterial infection. While caries scores are significantly increased in Aqp5-deficient mice, there is not an overwhelming enhancement in caries levels as seen in desalivated mice. Clearly, the organic constituents of saliva also provide protection against caries, even at markedly reduced salivary flow (60 to 65%) [13]. This scenario is possibly what happens in humans. We hypothesize there is great variation in levels of salivary flow in the population, as well as in the composition of the saliva, and groups with higher caries experience may be over-represented by individuals with small but significant decreases in salivary flow related to decreased *AQP5* activity due to genetic variation. These decreased levels of salivary flow may not be dramatic enough to have obvious clinical consequences or be related to direct patient complaints. Our group and others have shown that genes play a role in the host susceptibility component of the caries pathogenesis [14–32]. Wang et al. [29] studied two genetic variants in the aquaporin locus 12q13, rs923911 in *AQP5* and rs1996315 in *AQP6*. The authors reported association with the marker located in *AQP6*, suggesting it would have a protective effect on caries experience. In the present study, aquaporin genetic variations were investigated in regards to their potential role in caries, as well as the potential mechanism of *AQP5* that impacts the disease.

## Materials and Methods

### DNA Samples

Unstimulated saliva samples were obtained from all participants (subjects were asked to spit) and stored in Oragene DNA Self-Collection kits (DNA Genotek Inc) at room temperature until being processed. No centrifugation was performed in the saliva samples. No plaque samples were collected. DNA was extracted according to the manufacturer’s instructions. The only exception was the group from Brazil, which DNA was extracted immediately after saliva collection, based on a published protocol [33]. DNA samples from 1,383 subjects from five study groups analyzed sequentially were used in this project. These groups are summarized in Table 1.

**Table 1:** Summary of all individuals analyzed in tests of association, gene expression, and enamel microhardness, and their genotypes*.

	North American	Turkish (Istanbul)	Argentinean	Turkish (Marmara)	Brazilian	Turkish (Enamel Microhardness)
Sample size (mean DMFT^a^ ± SD^b^)	318 (15.4 ± 8.4)	172 (3.8±4.0)	274 (7.1 ± 7.8)	259 (5.2 ± 5.5)	359 (21.8 ± 7.6)	100 (5.2 ± 3.4)
Females	174	93	83	130	257	62
Males	144	79	60	129	105	38
Age (mean ± SD)	45.6 ±17.6	5.4 ± 0.8	21.7 ± 15.6	4.6 ± 0.6	55.8 ± 12.5	17.2 ± 3.1
The number of pedigrees	unrelated	unrelated	76	unrelated	unrelated	Unrelated
rs461872 (AA/AG/GG)	58/97/76	54/77/43	113/129/65	44/116/88	51/149/155	Not genotyped
rs467323 (AA/AG/GG)	117/102/46	24/81/59	92/126/36	55/125/77	95/156/103	16/51/33
rs10875989 (CC/CT/TT)	26/97/148	64/84/35	48/115/100	57/120/80	57/160/138	14/55/31
rs2878771 (CC/CG/GG)	10/90/199	11/71/93	2/42/219	29/112/116	108/102/146	7/34/56
rs3759129 (AA/AC/CC)	215/75/12	130/43/0	196/38/11	184/58/5	248/85/16	Not genotyped
rs1996315 (CC/CT/TT)	62/120/70	58/82/23	53/135/70	31/133/89	69/174/107	1/17/71
rs3741559 (AA/AG/GG)	10/70/181	102/62/4	194/37/3	124/108/26	143/102/104	Not genotyped
rs296763 (CC/CG/GG)	18/92/143	0/49/109	3/71/186	2/67/183	19/118/216	2/33/65

^a^ Decayed, Missing due to caries, Filled Teeth

^b^ Standard Deviation

* Totals may not match with sample sizes due to PCR failure.

Pittsburgh, USA. DNA samples of 318 subjects from the University of Pittsburgh School of Dental Medicine Dental Registry and DNA Repository (DRDR) were studied first. The DRDR protocol is approved by the University of Pittsburgh Institutional Review Board (IRB# 0606091) and all subjects provided written informed consent prior to participation. Parents or legal guardians provided written inform consent for minors and an assent document was used for all children 7 years of age or older. The mean age of subjects was 43.23 years with ages ranging from 17 to 84 years.

Istanbul University, Istanbul, Turkey. DNA samples of 173 children recruited as approved by both the Istanbul University (IRB#2006/2508) and University of Pittsburgh (IRB # PRO07100045) Institutional Review Boards (IRB) were used to confirm initial results of Pittsburgh. Written informed consent was obtained from all parents of participant children. Eligible children were from 3 to 6 years of age and were enrolled in the Pedodontics clinics of Istanbul University and daycare facilities in the city of Istanbul.

Patagonian region, Argentina. DNA samples from 274 individuals comprising 76 families (143 individuals were unrelated) living in twelve Patagonia sites were studied (San Carlos de Bariloche, El Bolsón, Esquel, El Maitén, Maquinchao, Ingeniero Jacobacci, Rio Colorado, Choele Choel, Valcheta, Sierra Grande, Santo Antonio Oeste, and General Roca). The mean age was 21.7 years (between infants under 1 and 72 years with median of 18 years) and both the Centro de Educación Médica e Investigaciones Clínicas “Norberto Quirno” (CEMIC) (IRB#543) and University of Pittsburgh (IRB#405013) Institutional Review Boards approved the study of these samples and appropriate written informed consent was obtained from all participants (parents provided consent for the participation of individuals 17 years of age and under). For this particular study group, saliva samples were collected twice from each participant. One was used for DNA extraction, and the second was used for RNA extraction. Oragene RNA Self-Collection kits (DNA Genotek Inc) were used for the second saliva sample and these samples were also kept at room temperature until being processed. No centrifugation was performed in the saliva samples.

Marmara University, Istanbul, Turkey. DNA samples from 259 children recruited as approved by the Human Ethics Committee of Marmara University (IRB#06.01.2012–5), and the University of Pittsburgh IRB (IRB # PRO07100045) were also studied. Written informed consent was obtained from all parents of the participant children. All participants were unrelated systemically healthy children from 2 to 5 years of age whose parents denied systemic fluoride consumption, seeking dental treatment at the Pediatric Dental Clinics of Marmara University.

Niterói, Brazil. Finally, 359 DNA samples from individuals recruited as approved by the Research Ethics Board at the Fluminense Federal University, Niterói, Rio de Janeiro state (CEP CMM/HUAP 070/07 under protocol CAAE 0061.0.258.000–07), and University of Pittsburgh IRB (IRB#PRO012080056) were studied. Written informed consent was obtained from all participants. The mean age of subjects was 55.8 years with ages ranging from 41.4 to 67.7 years.

### Caries Experience

Caries experience data (DMFT/dmft) were collected according to a modification of the World Health Organisation protocol of 1997 [34]. In brief, visible lesions in dentin, as well as visible active lesions in enamel (white spots) were scored as decayed. An explorer was gently used for assessing the smoothness of tooth surfaces. Exam calibrations were possible in Brazil and Turkey and were performed according to the following protocol: First, the calibrator presented to the examiner(s) the criteria for caries detection, showing pictures of several situations to be observed in the exam (sound and decayed tooth surfaces, filled teeth with and without secondary lesions, missing teeth due to caries or due to other reasons) and discussing each of these situations in a session that lasted one to two hours. Next, the calibrator and examiner(s) examined 10 to 20 subjects and discussed each case.

Pittsburgh. Data from the DRDR were extracted from the dental records by one of the coauthors (I.A.) after the review of chart notes and radiographs. No exam calibration was possible. Aside from DMFT/dmft scores, DMFS/dmfs scores were also obtained. Water is Pittsburgh is fluoridated and subjects from the DRDR are commonly affected by the most common adult diseases [35]. Since saliva flow is an important parameter related to the possible genetic influence of aquaporin, we defined two variables for this population group to serve as a proxy for direct assessment of saliva flow. “Saliva flow” was defined based on the amount of saliva subjects spit during the saliva sample collection for DNA extraction (in general patients take 5 minutes to provide 4.0 milliliters of saliva). Any subject that spit less than 3.0 milliliters was considered as having low salivary flow. Also, the reported medications taken by each subject was used to categorize him or her into two groups: Using medications that cause decreased salivary flow (or dry mouth) as a reported side effect (the list of medications we used as reference is presented as a supplement; S1 Table), and using medications with no side effects affecting salivary flow. Individuals using no medications were grouped with the individuals that have medications causing no salivary flow reduction. These two variables were used to adjust the genetic association analysis.

Istanbul University, Istanbul, Turkey. In the case of data from University of Istanbul, one of the authors (A.P.) carried out the clinical examination after being calibrated by an experienced specialist (F.S.). The intra-examiner agreement was assessed by a second clinical exam in 10% of the sample after 2 weeks, with a κ of 1.0. Differently from the rest of the groups, preference was given to children with four or more decayed or filled tooth surfaces, or children with no evidence of caries (including no evidence of white-spot lesions), and no history of caries. Radiographs were available for all participants and dmfs scores were also generated. Subjects with dmfs > 0 but < 4 were not included in the study. Most parents reported not brushing the teeth of their children. Drinking water in the region is not artificially fluoridated.

Patagonian region, Argentina. In the case of Argentina, data were collected by one single experienced specialist examiner (A.R.V.). Subjects in these projects are seen over a period of no longer than three to five days and intra-examiner agreement data could not be generated. No radiographs were available but drinking water in some sites has a high concentration of fluoride and many subjects had moderate to severe fluorosis.

Marmara University, Istanbul, Turkey. Subjects from the Marmara University were examined by one of the coauthors (Z.A.), after calibration by an experienced specialist (A.M.K.). Radiographs were available and both dmft and dmfs scores were obtained. Subjects with dmfs = 1 were not included in the study. The intra-examiner agreement was assessed by a second clinical exam in 10% of the sample after 2 weeks, with a κ of 1.0.

Niterói, Brazil. Subjects from Niterói, Brazil, were examined by one of the coauthors (P.L.C.). Since this cohort was older than the previous ones studied, we defined two discreet groups of caries experience (low and high caries experience) based on age for analyses (Table 2).

**Table 2:** Criteria used to define caries experience in the samples from Niterói, Brazil.

Criteria		
Caries Experience Level	DMFT	n
Young Adults [from 23 to 39 years of age]		
Low caries experience	0–8	15
High caries experience	9 or higher	15
Middle age [from 40 to 59 years of age]		
Low caries experience	0–19	84
High caries experience	20 or higher	117
Elderly [60 years of age and older]		
Low caries experience	0–21	47
High caries experience	22 or higher	81

### Single Nucleotide Polymorphism (SNP) Genotyping and Association Analysis

**Fig 1:**
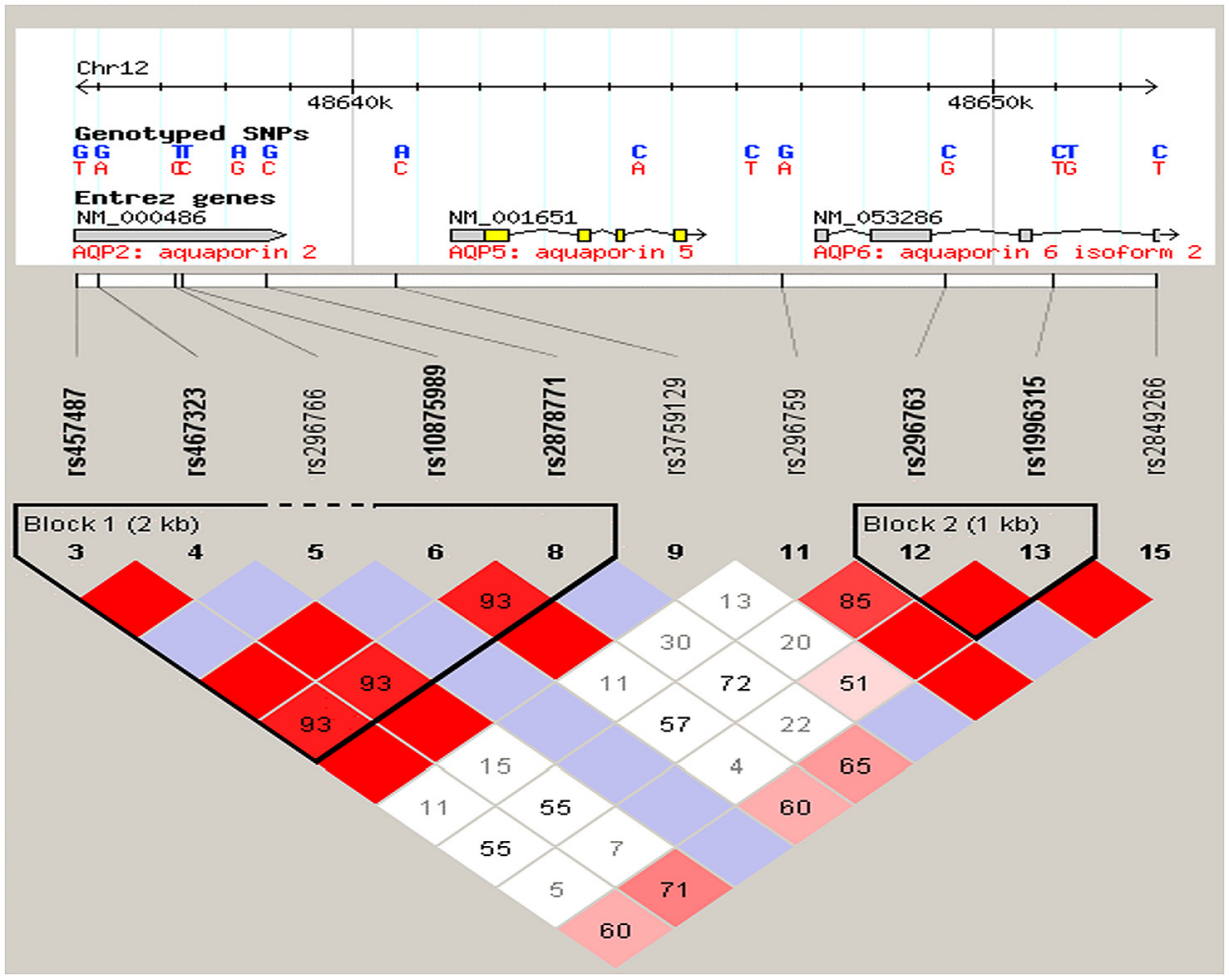
Linkage disequilibrium plot illustrating the aquaporin locus on 12q13 region investigated. The single nucleotide polymorphisms are located in their approximate geographic positions in the chromosome and are distributed among the three genes. The relationship between two SNPs is represented by the intersection between the two squares and may present different colors (or color intensity) based on the value obtained for each pair. Red indicates D’ = 1 and LOD ≥ 2. Blue indicates D’ = 1 and LOD < 2. Shades of red/pink indicate D’< 1 and LOD ≥ 2. White indicates D’< 1 and LOD < 2. Markers selected for this studied include rs457487, rs467323, rs10875989, rs2878771, rs3759129, rs296759, rs296763, and rs1996315.

In order to select markers for the *AQP5* gene we used data from the International HapMap Project (haplotype map of the human genome). Single nucleotide polymorphisms (SNPs) were selected based on minor allele frequency, the gene structure and linkage disequilibrium relationships between SNPs (Fig 1). We used the approach devised by Carlson et al. [36], selecting from a set of SNPs that maximally represent the linkage disequilibrium structure of a given region.

Eight selected SNPs (Fig 1, Table 1) were genotyped using Taqman chemistry [37] on an automatic sequence-detection instrument (ABI Prism 7900HT, Applied Biosystems, Foster City, CA). Assays and reagents were supplied by Applied Biosystems (Applied Biosystems, Foster City, CA).

Hardy-Weinberg equilibrium tests were performed to test for deviations in the genotype and allele distributions. We used logistic regression analysis (as performed by the PLINK analysis software [38]) to investigate main-effect models to predict caries status. All analyses were adjusted by age and sex, and the data from Pittsburgh was also adjusted by salivary flow and the use of medications that cause dry mouth. A p-value ≤ 0.05 was considered nominally statistically significant, with a p-value lower than 0.006 significant after correction for multiple comparisons. The FBAT package [39] was used to analyze the data from Argentina. These analyses were done with all the families combined, as well as subdivided by families from sites where moderate to severe fluorosis was identified and by sites without clinical fluorosis. P-values 0.05 or lower were considered statistically significant. Both single marker and haplotype analyses were performed.

### Mutation Analysis

We carried out mutation analysis on the aquaporin genes in 12q13 in 322 DNA samples from unrelated individuals (96 from Pittsburgh, 173 from Istanbul, Turkey, and 53 from Argentina). These genes included *AQP2*, *AQP5*, and *AQP6*. Reference sequences were obtained from the Ensembl Genome Browser (http://useast.ensembl.org/index.html). The primers used for PCR amplification are available upon request. DNA was amplified by 35 cycles of at 95°C for 30 seconds, 55°C for 30 seconds, and 72°C for 1 minute and PCR products were directly sequenced using an ABI PRISM BigDye^™^ Terminator Cycle Sequencing Ready Reaction Kit and an ABI 3730xl DNA Analyzers (Applied Biosystems, CA, USA). Sequences obtained were verified against the sequences in the Ensembl Genome Browser and two unrelated CEPH (Foundation Jean Dausset-Centre d'Etude du Polymorphisme Humain) controls.

### Gene-Gene Interaction Analysis

The evidence that AQP5 is expressed in the dental lamina, inner enamel epithelium, stratum intermedium, stellate reticulum and the outer enamel epithelium [5], although the amount of observed expression in these different structures depend on the tooth germ being on the early versus the late bell stage, suggest a possible mechanism of AQP5 in caries is related to the characteristics of the enamel structure and how susceptible this structure is to the carious attack. To generate data that may support this hypothesis, we tested if there is statistical evidence that *AQP5* alleles are associated with caries experience depending on genetic variation in genes reported to contribute to the formation of enamel formation (ameloblastin, amelogenin, enamelin, tuftelin, and tuftelin interacting protein 11). Genotypes for these genes were generated in markers used in our previous studies [15, 16, 24, 40] in the Turkish data set from the Marmara University according to the description above. Logistic regression was used to test for association between *AQP5* alleles and caries experience, depending on genetic variants in enamel formation genes. P ≤ 0.05 were considered statistically significant.

### mRNA expression analysis of *AQP5*

Whole saliva RNA samples were obtained from 152 individuals from the Argentinean population described above and total RNA from saliva was isolated using RNeasy Micro kit (Qiagen, CA, USA). mRNA expression analysis of *AQP5* was performed. Total RNA from the submaxillary salivary gland epidermoid carcinoma cell line HTB-41^™^ and from the mammary gland/breast adenocarcinoma cell line HTB-22^™^ (American Type Culture Collection, Manassas, VA, USA) were also isolated and studied. This breast cancer cell line was chosen since it is known to express *AQP5*. 100 ng of total RNA from whole saliva was reverse-transcribed with the High Capacity cDNA Reverse Transcription kit (Applied Biosystems, CA, USA). The primers used for the RT-PCR and real-time PCR are available upon request. *β-Actin* was used as endogenous control. For real-time PCR analysis, PCR amplification of cDNA was performed by SYBR Green PCR Master Mix (Applied Biosystems, CA, USA). Products spanned at least one intron, so that cDNA products were distinguishable from potential genomic DNA products. After an initial denaturation at 95°C for 5 minutes, 40 cycles at 95°C for 45 seconds, 55°C for 45 seconds, and 72°C for 90 seconds were performed in a 7900HT Real-time PCR system. The quantification of mRNA expression levels relative to *β-Actin* was performed by 2DDCT method [41]. The Student’s *t* test was used to analyze differences based on caries experience, age, sex, and living in an area with high fluorosis experience. P-value ≤ 0.05 was considered statistically significant.

To validate the mRNA data from the experiment in human saliva, we compared mRNA expression levels with protein levels of AQP5 in eight healthy adult individuals without any caries activity. After generating mRNA data as described above, the same saliva samples had their proteins extracted. Proteins were transferred to PVDF nylon membranes and exposed to COOH-terminal-specific anti-AQP5 antibody (Santa Cruz Biotechnology, Inc., Santa Cruz, CA, USA). Alkaline phosphatase conjugated secondary antibodies were used and detection of antibody binding was carried out using alkaline phosphatase detection kit (Vector Laboratories, Burlingame, CA, USA) [42].

### *AQP5* Expression in Cells Exposed to High Concentrations of Fluoride

**Fig 2:**
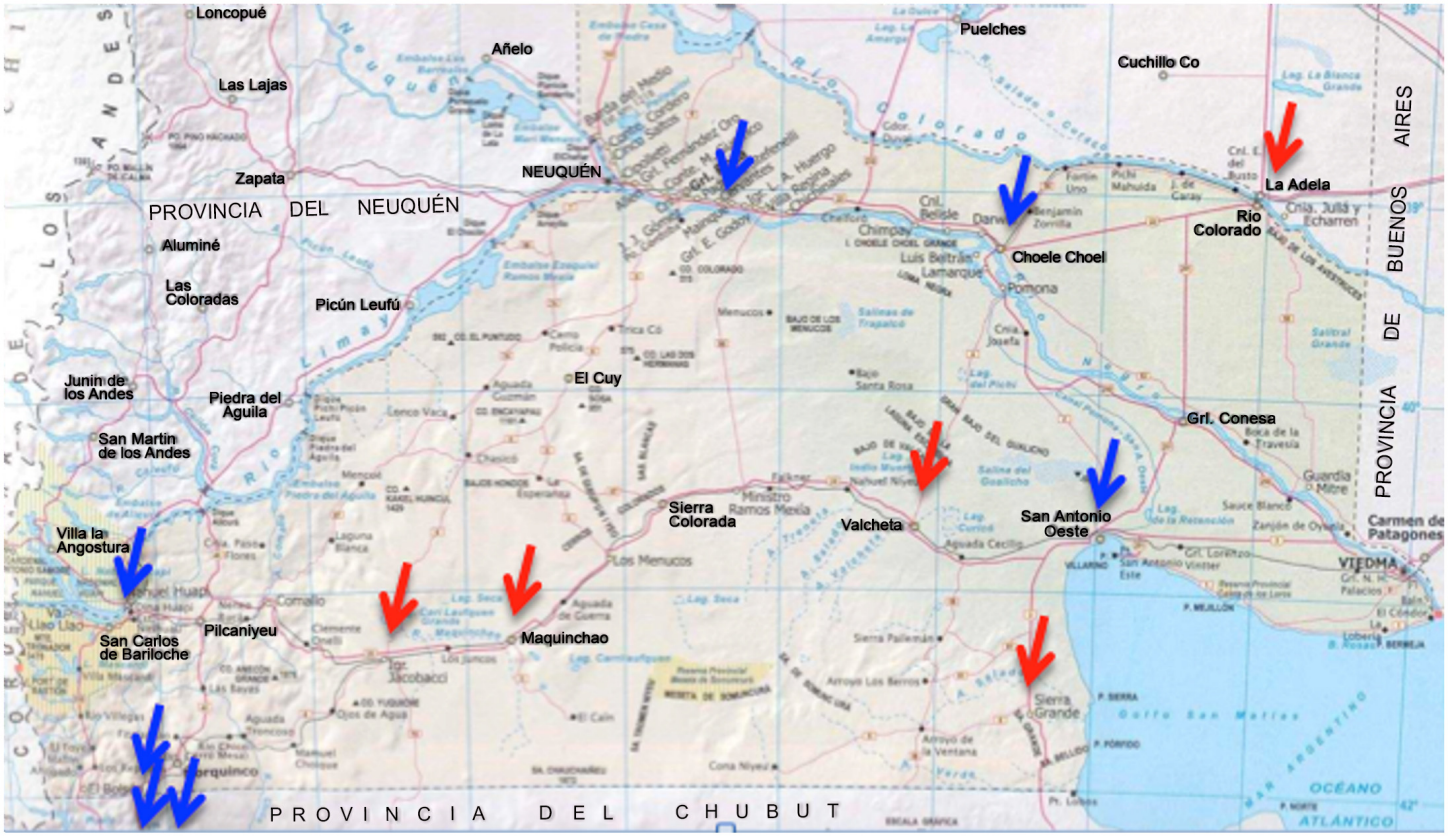
Arrows indicate sites where saliva samples were collected in Argentina. Red arrows indicate sites with very high frequency of cases with moderate to severe fluorosis and blue arrows sites where virtually no fluorosis cases were found.

The mammary gland/breast adenocarcinoma cell line HTB-22^™^ (American Type Culture Collection, Manassas, VA, USA) was cultured and incubated to mimic increased levels of fluoride exposure, similar to the levels found in the Argentinean region (up to 10 ppm in parts of the country with mean levels of 3.2 (standard deviation 2.7) [43,44], Fig 2 shows the sites where clinical fluorosis was found in our study). This cell line was chosen since it is reported to express AQP5. We also attempted to perform these experiments in the submaxillary salivary gland epidermoid carcinoma cell line HTB-41^™^ (American Type Culture Collection, Manassas, VA, USA), however we did not detect any evidence of AQP5 expression in these cells.

Five cell cultures were established. Each culture was incubated for 20 hours with NaF (Thermo Fisher Scientific, Beverly, MA, USA) for 20 hours at concentrations of 1ppm (50μM), 2ppm (100μM), 4ppm (200μM), and 5ppm (250μM) of F. One culture was not exposed to NaF and served as reference. The expression of AQP5 in cultures treated with various concentrations of NaF was compared by means of ANOVA with the Tukey *post hoc* test. Statistical significance was defined as p-value ≤ 0.05.

### *Aqp5* Expression in Wistar Rats Exposed to Fluoridated Water

Male weanling Wistar rats were distributed into three groups containing 9 animals each that differed in respect to the fluoride concentration in the drinking water (control that received deionized water, 5 ppm F or 50 ppm F, as NaF). The animals were housed in groups of three per cage into climate-controlled room at 21.0 ±3°C with 55±5% relative humidity under 12 hours light/dark cycle, and received food and water *ad libitum* for 60 days. At the end of the experimental period the animals were anaesthetized with ketamine and xylazine and parotid glands were collected and flash frozen. All experimental protocols were approved by the Ethics Committee for Animal Experiments of Bauru Dental School, University of São Paulo (Protocol 31/2010).

Western blots were performed as previously described [45]. Submandibular glands were removed and homogenized in RIPA buffer (150 mM sodium chloride, 0.5% sodium deoxycholate, 0.1% SDS, 1% NP-40, 50 mM Tris) supplemented with protease inhibitors (Roche Diagnostics, Mannheim, Germany). After removal of the tissue debris by centrifugation at 800 g for 15 minutes at 4°C, protein concentrations were determined by Quick Start^™^ Bradford (Bio-Rad, Hercules, USA) [46]. Protein samples (40 μg) were resolved by 15% sodium dodecyl sulfate-polyacrylamide gel electrophoresis and transferred to polyvinylidene fluoride membranes. For immunoblotings, membranes were incubated with primary antibodies anti-aquaporin 5 (1:5000) (Millipore, USA) and anti-α-Tubulin (1:5000) antibodies (Abcam ab52866) for 2 hours at room temperature followed 1 hour with HRP-linked secondary antibodies (1:2000) (GE healthcare, Buckinghamshire, UK). Blots were developed using ECL Prime (GE healthcare, Buckinghamshire, UK) detection reagents and then exposed to X-ray film. Blots films were digitalized with ImageScanner II (GE Heathcare, Uppsala, Sweden) and density was determined by Image J software (NIH, USA). Relative density was obtained after normalization with GAPDH, and the data were submitted to one-way analysis of variance (ANOVA) followed by Bonferroni *post-hoc* tests performed by GraphPad Prism 5 (GraphPad Software, San Diego, USA).

### Enamel Microhardness and Association with Aquaporin Locus Genetic Variants

Enamel samples from extracted premolar teeth from 100 orthodontic adolescent patients (63 with high dental caries experience and 37 with low caries experience) from Istanbul University were used in enamel microhardness testing (Table 1). The enamel samples came from premolars and were used to test the association between genetic variation in *aquaporins* and enamel microhardness at baseline, after simulating artificial caries, and after fluoride treatment. The goal was to test the hypothesis that *aquaporin* influences dental caries by generating a more susceptible enamel surface to acidic dissolution. The Ethics Committee of Istanbul University approved this study, and informed consent from all participating patients was obtained. Subjects age ranged from ten to 32 years (mean age of 17.2 years; 38 males and 62 females). The mean DMFT ranged from zero to 17 (mean DMFT 5.2; 63 with high caries experience and 37 with low caries experience). Tooth samples were cleaned of any remnants and stored in a 10% formalin solution (pH = 7.0) at room temperature until the initial polishing. The crowns of each tooth were separated from the roots and then separated again buccolingually and mesiodistally. The five surfaces studied were occlusal, mesial, buccal, distal, and lingual. The surfaces were sanded for one minute, at a force of 1 lbf, while moving at a speed of 20 rpm on paper of 320, 400, and 600 grit, and then polished for seven minutes at a force of 1 lbf at a speed of 25 rpm in 6μm, 1μm, and 0.25μm diamond suspension. Sample baseline microhardness was tested using a microhardness tester (IndentaMet 1100, Buehler Ltd.) with a knoop diamond. Five indentations under a load of 25 grams for five seconds were made. Next, artificial caries was simulated by immersing the samples in 24 mL of demineralizing solution (1.3 mmol/L Ca, 0.78 mmol/L P, 0.05 mol/L acetate buffer, 0.03 μg F/mL, pH:5.0) at 37°C for 16 hours. Microhardness was again measured by five indentations created just below the initial ones. These indentations were then exposed for ten minutes to a fluoride solution, created from toothpaste containing sodium fluoride (1,400 ppm fluoride), to determine if microhardness for the artificial caries lesions would be brought back to baseline levels after fluoride exposure. Again, surface microhardness was measured with five more indentations below the previous ten.

The results of the microhardness testing were compared to the genotyping of DNA extracted from saliva from each of the 100 patients. Results were analyzed using the PLINK software package. [38] Mean microhardness at baseline, after artificial caries lesion creation, and after fluoride application was calculated. Subjects were divided into two comparison groups: above and below the means. We made comparisons by surface, as well as by using the mean enamel microhardness of all five surfaces combined. A p-value of 0.05 was considered statistically significant.

## Results

### Single Nucleotide Polymorphism (SNP) Genotyping and Association Analysis

All genotypes (Table 1) were in Hardy-Weinberg equilibrium. Multiple nominally significant associations were found between all population groups and markers in the *AQP5* locus. In the sample from Pittsburgh, the best model showed that higher caries experience is influenced by a combination of older age, use of medications that cause xerostomia and genetic variation in *AQP5* SNPs rs3759129 (p = 0.03) and rs10875989 (p = 0.04). Since the influence of age on caries experience in this group was marked, we also tested a pediatric case-control cohort from Istanbul University, Turkey. This analysis confirmed an association between higher caries experience and rs3759129 (p = 0.03). The analyses of the cohort of families from Argentina only revealed associations when families from sites with clinical fluorosis were excluded from the analyses (Fig 2). The haplotypes of the *AQP5* locus SNPs rs461872-rs1996315 under a dominant model (p = 0.03), and rs461872-rs1996315 (p = 0.05) and rs3759129-rs1996315 (p = 0.01) under a recessive model showed association with higher caries experience. The additional analysis of the second cohort from Turkey (Marmara University) showed an association between rs467323 and higher caries experience (p = 0.03). Finally, the sample from Brazil showed an association between higher caries experience and rs10875989 (p = 0.01 under a recessive model).

### Mutation Analysis

To test the hypothesis that a variant in the coding region of *AQP2*, *AQP5*, or *AQP6* in linkage disequilibrium with the studied markers contributed to caries, we performed sequencing of all exons and exon-intron boundaries of these three genes but no coding variants were identified, despite some non-synonymous variants are reported in public databases. Although *AQP2* and *AQP6* are not know for being expressed in the salivary glands aside from the kidneys, these genes were included in the analysis since they are in close proximity to *AQP5*.

### Gene-Gene Interaction Analysis

We found statistical evidence for interaction between a marker in *AQP5* (rs3759129) and rs3759129 in tuftelin (p = 0.02).

### mRNA expression analysis of *AQP5*

**Fig 3:**
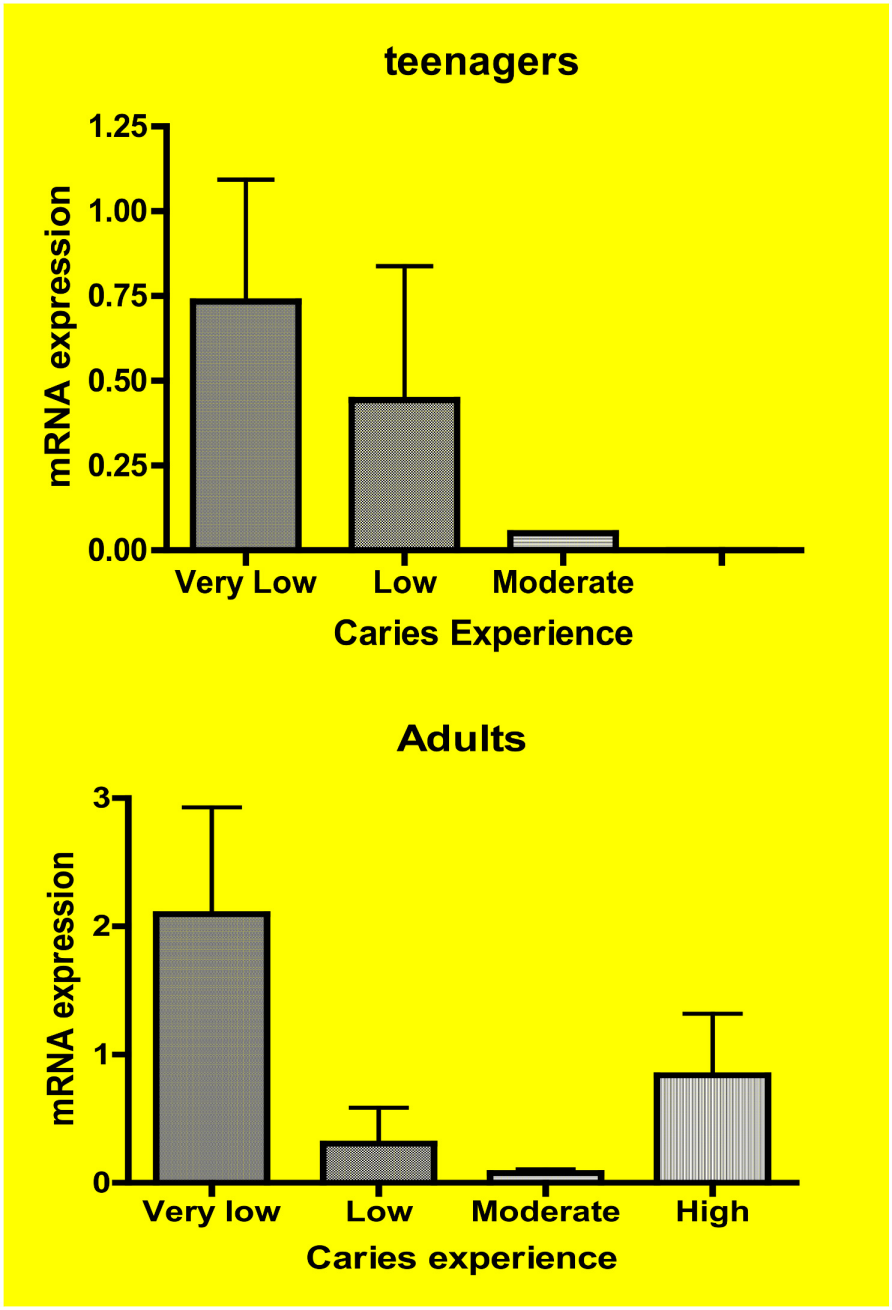
*AQP5* expression in whole saliva based on caries experience in teenagers (above) and adults (below). The expression is higher when individuals have lower caries experience. The increased expression in adults classified as high caries experience likely corresponds to lack of caries activity as assessed by the increased number of missing teeth rather that active decayed teeth. Caries experience was defined as “Very Low,” “Low,” “Moderate,” and “High” according to our previous work [17]. These discreet groups include caries experience levels taking into consideration ages, since DMFT scores tend to increase in individuals as they get older.

**Fig 4:**
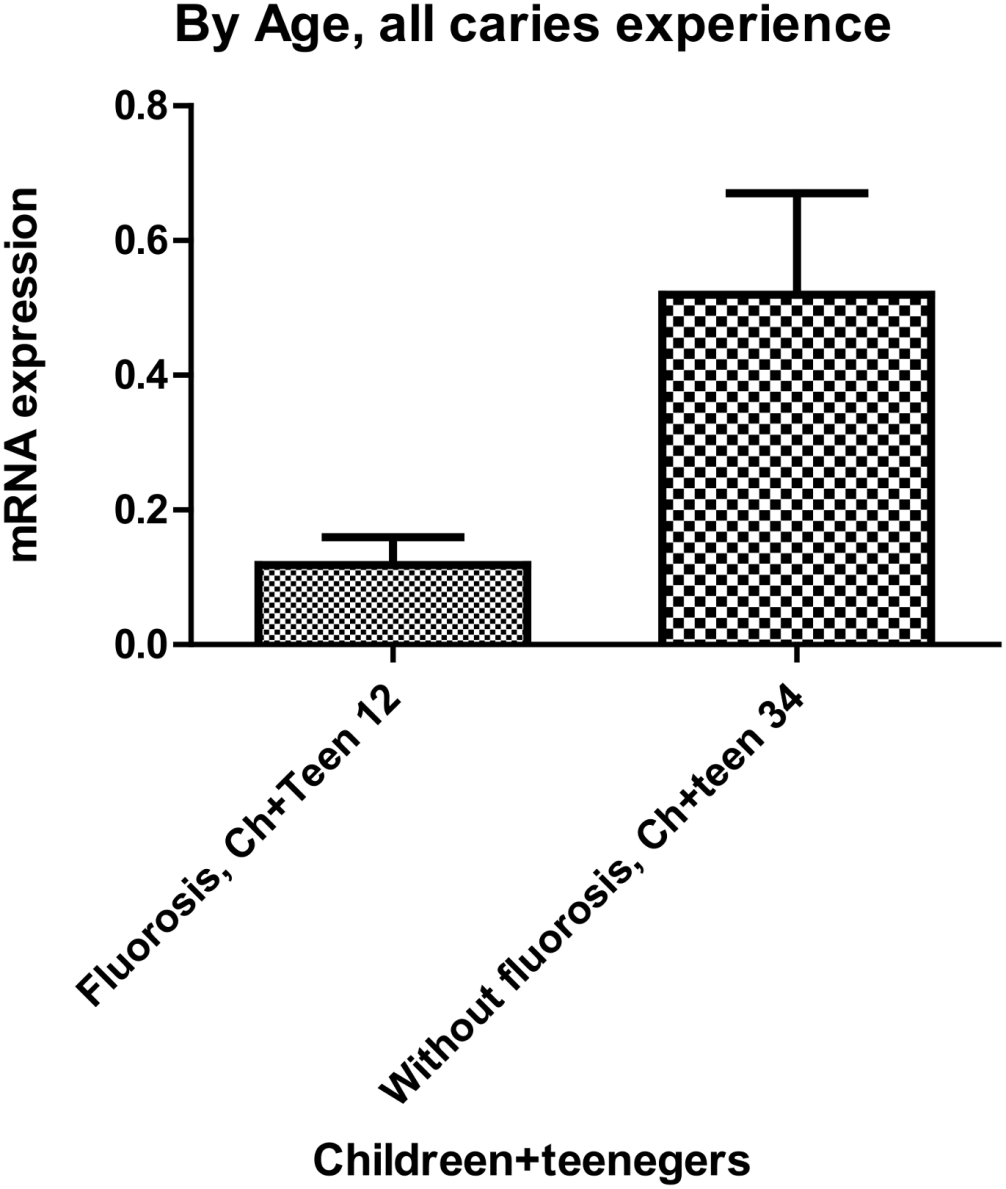
*AQP5* expression in whole saliva is lower [N = 12 children (ch) and teenagers (teen)] in individuals with moderate to severe fluorosis [N = 34 children (ch) and teenagers (teen)].

**Fig 5:**
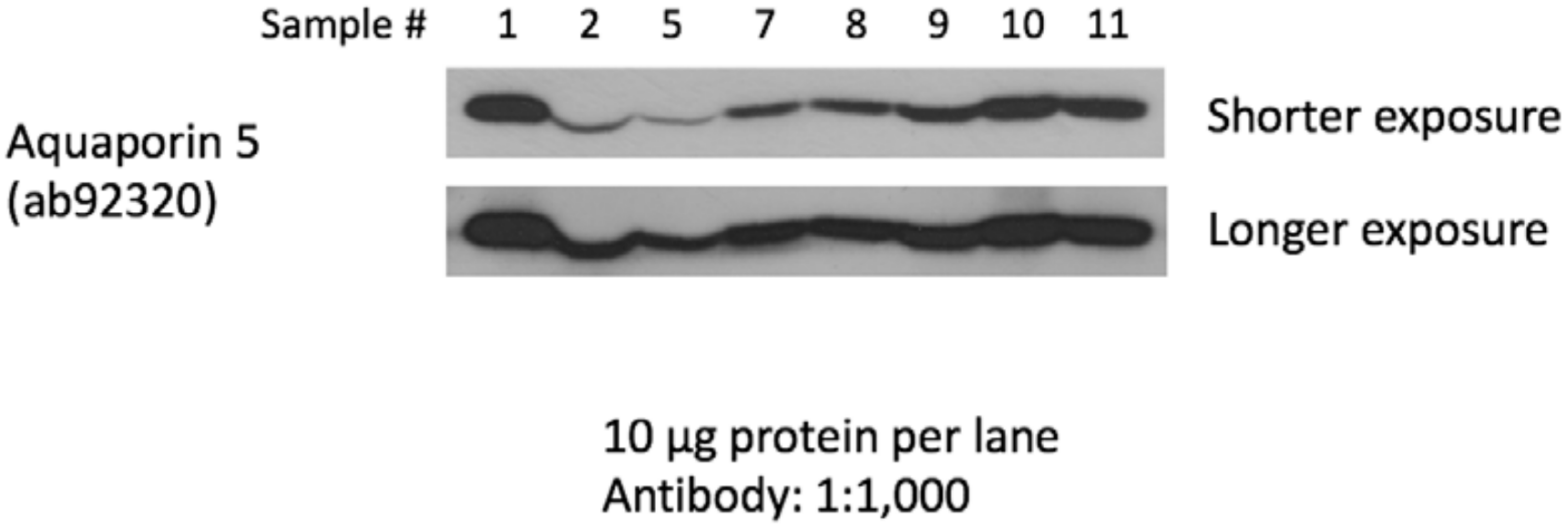
Immunoblot analysis of saliva samples from eight healthy adults without any caries experience demonstrated that AQP5 can be detected both by quantitative real time PCR as well as western blotting (all eight samples had expression levels of *AQP5* similar to the samples with low caries experience from the Argentinean cohort.

*AQP5* expression is higher in whole saliva of individuals older than 12 years of age with low caries experience (p = 0.03; Fig 3). *AQP5* expression in whole saliva of individuals from areas with fluorosis is decreased (p = 0.04; Fig 4). The detection of *AQP5* mRNA in whole saliva of healthy adults without caries activity correspond to the presence of the protein (Fig 5).

### *AQP5* Expression in Cells Exposed to High Concentrations of Fluoride

**Fig 6:**
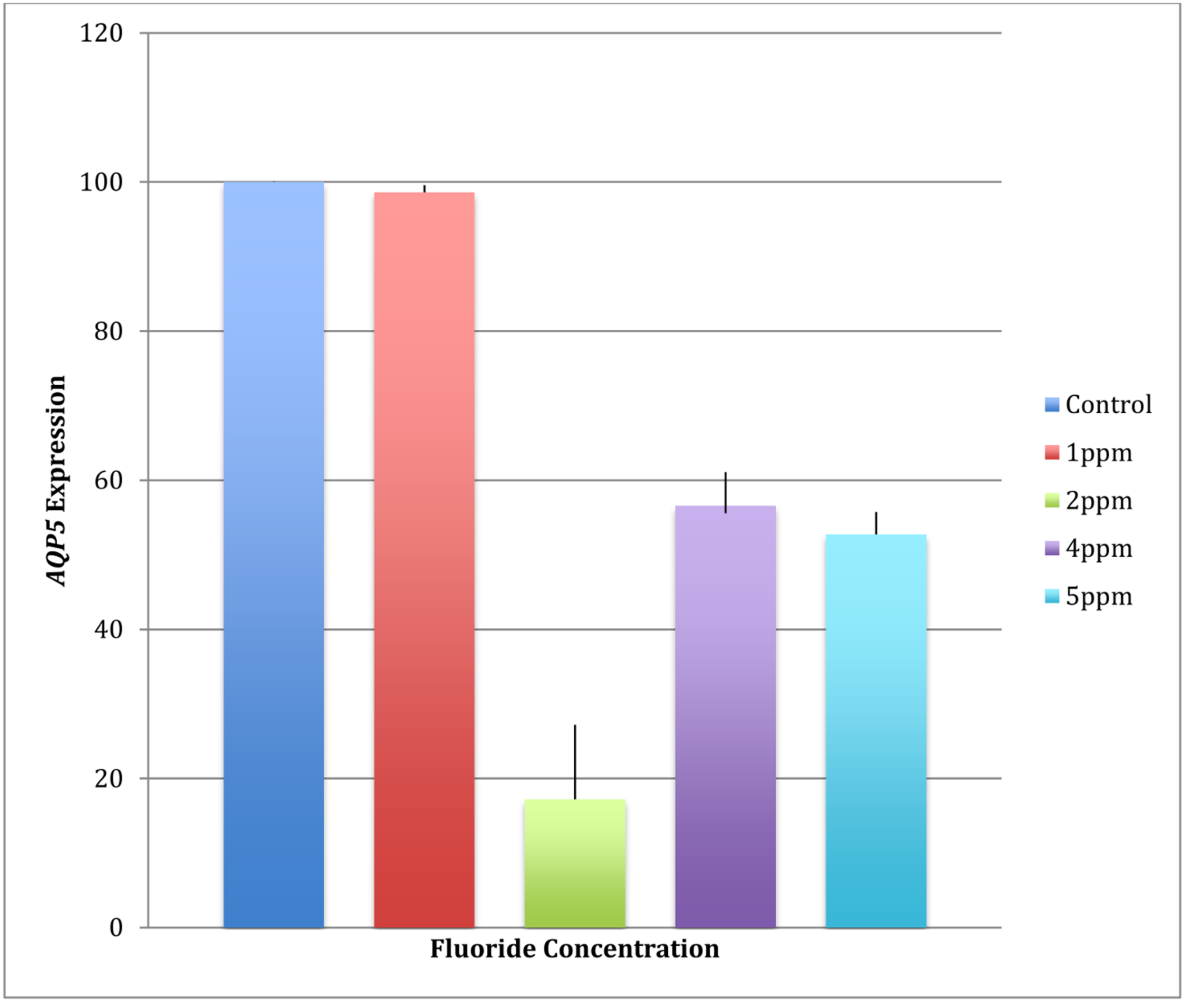
*AQP5* expression in mammary gland/breast adenocarcinoma cell line HTB-22^™^ after exposure to higher concentrations of fluoride decreases.

*AQP5* expression in mammary gland/breast adenocarcinoma cell line HTB-22^™^, a cell line known to express *AQP5*, decreased 40% to 80% with exposure to higher concentrations of fluoride compatible to clinical fluorosis in humans (Fig 6). The data also suggest that this decline in AQP5 expression is not dose dependent.

### *Aqp5* Expression in Wistar Rats Exposed to Fluoridated Water

**Fig 7:**
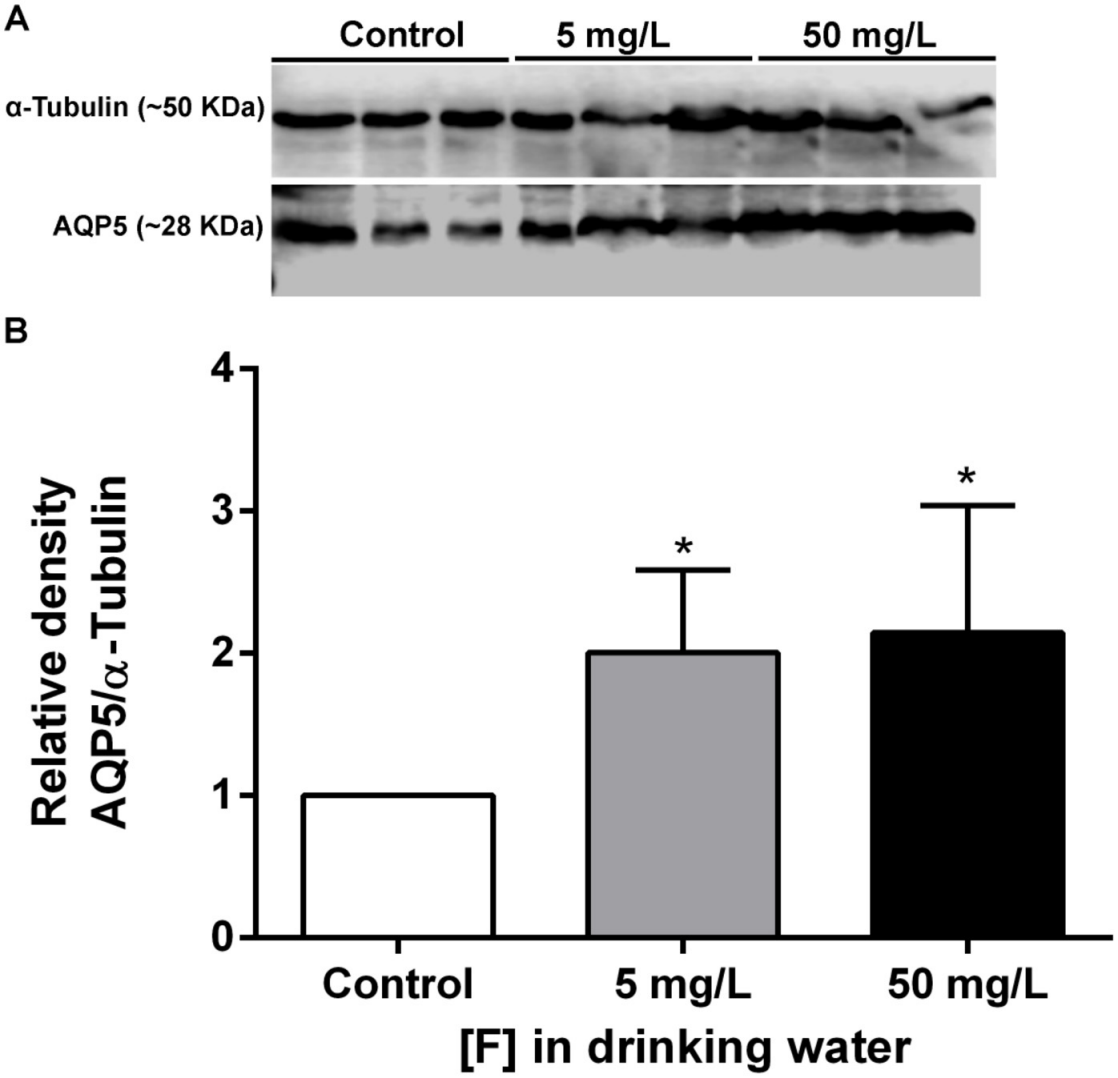
Effect of chronic exposure to F in Aqp5 expression in the submandibular glands of rats. (**A**) Representative blotting of Aqp5 (~28 kDa) and loading control α-Tubulin (~50 kDa). (**B**) Relative densitometric analysis of Aqp5 protein normalized with α-Tubulin. The expression of Aqp5 was significantly higher in fluoride exposed groups when compared with control (*p<0.05). Values are mean ± SD of three independent experiments.

Fluoridated water at optimal level (1 ppm in humans, which is equivalent to 5 ppm for rats) increases the expression of Aqp5 in the submandibular gland of rats (Fig 7).

### Enamel Microhardness and Association with Aquaporin Locus Genetic Variants

We found no evidence of association between genetic variants in the aquaporin locus and enamel microhardness. Enamel microhardness data are available as a supplement, S2 Table.

## Discussion

Studies of the human red blood cell RH protein led to the discovery of a water channel protein, aquaporin 1 (AQP1). Transfer of AQP1-injected *Xenopus laevis* oocytes to hypotonic solution led to swelling and rupture, which provided evidence that AQP1 was a water-specific channel [47]. Two decades after of this discovery, the first human trials of adenoviral-mediated transfer of AQP1 cDNA for radiation-induced salivary hypofunction are showing subjective improvement in xerostomia [48]. It turns out AQP1 is required for the kidney to concentrate urine, but it is absent in glandular epithelia. AQP5 provides the pathway for water secretion of lacrimal and salivary glands. Salivary glands consist of secretory lobules with serous- and mucous-secreting cells and excretory ducts lined with a distinct epithelium that modifies the basal cell secretions. Neural and hormonal stimuli induce large and rapid reductions in the cell volume of salivary acinar cells [49]. The potential of modulating saliva function made us hypothesize that genetic variation in *AQP5* contributes to the pathogenesis of caries. In the present study, we designed a series of experiments to demonstrate that an association between caries experience and *AQP5* genetic variation and to explore the possible mechanisms of *AQP5*’s contribution to caries risk.

Our genetic association studies suggested there is an association between caries experience and genetic variation in or flanking *AQP5*. These results confirm a previous study done in North Americans [29] but demonstrate how challenging it is to interpret such results. We cannot find positive association results for the same genetic markers, likely due to differences in allele frequencies, phenotype definitions, statistical power, and demographics of the populations studied. Hence, these studies provided us with two notable clues:

1) The evaluation of adults from Pittsburgh suggested that studying adults was likely confounded by the influence of age in the caries experience of the population. We decided to eliminate the age effect by studying a much younger group with primary dentition where the controls were older than the cases. These analyses suggested that the same marker in *AQP5* is associated with high caries experience in adults and in children; and, 2) The studies with the samples from Argentina, which included families recruited in sites where fluorosis is prevalent, allowed the evaluation of genetic associations based on the origin of the samples (*i*.*e*., samples from a site with fluorosis versus a site without fluorosis). Associations were found only when samples from sites where fluorosis can be found were excluded from the analysis. We hypothesized that the excess of fluoride interferes with the function of *AQP5*. We tested this hypothesis by three approaches. We evaluated the expression of *AQP5* in whole saliva from humans and demonstrated that the expression is lower in individuals from areas where fluorosis can be found. These expression levels correspond to the detection of the protein itself in saliva. We also found that *AQP5* expression was higher in individuals with lower levels of caries experience. When we exposed a cell-line reported to express AQP5 to high concentrations of fluoride, we demonstrated that AQP5 expression is the same at optimal levels of fluoride and decreases in a non dose-dependent manner in response to higher concentrations. Finally, we showed the opposite effect *in vivo*, when we exposed rats to optimal and higher levels of fluoride in the drinking water and showed that Aqp5 expression is higher at optimal and higher levels of fluoride in the water. This evidence suggests that optimal levels of fluoride in the drinking water may have the additional benefit of stimulating the expression of *AQP5*, which in turn may increase the protection against caries.

We also showed that *AQP5* may interact with proteins involved in enamel development, thereby impacting the formation of this structure and making it more or less susceptible to caries. However, we found no evidence that genetic variants in the aquaporin locus are associated with enamel microhardness, which suggests the most relevant contribution of *AQP5* to caries involves saliva function rather than influencing enamel development.

The importance of fluoride in preventing caries by favorably interfering in the demineralization-remineralization processes is well established. Fluoride has also an antimicrobial effect by inhibiting a number of bacterial enzymes, including enolase, an enzyme involved with substrate uptake and energy metabolism [50]. Another mechanism influencing caries progression in dentine involves matrix metalloproteinases (MMPs). We have shown that genetic variants in *MMP2* and *MMP3* are associated with periapical lesion formation in teeth with untreated deep caries lesions [51]. Further studies showed that MMP2 and MMP9 are inhibited by sodium fluoride (50–275 ppm of fluoride). This effect is irreversible at 5,000 ppm of fluoride [52]. Our study demonstrates for the first time an additional mechanism of fluoride that potentially impacts caries, which includes stimulating AQP5 expression in saliva.

In summary, we report evidence confirming previous suggestions that AQP5 is associated with caries experience in humans and propose that the mechanism for the possible influence of AQP5 in the pathogenesis of caries involve the impact on saliva function. We also showed evidence that AQP5 and fluoride interact and may act synergistically in certain individuals to protect them against caries.

## Supporting Information

S1 Table Medications that cause Xerostomia: http://www.drymouth.info/consumer/searchResults.asp. (DOCX)

S2 Table Summary Results of the enamel microhardness tests. DMFT/DMFS are the caries experience scores (Decayed, Missing due to caries, Filled Teeth or Surfaces). Each subject had one first premolar studied [mandibular (1st P.L) or maxillary (1st P.U)]. Know enamel microhardness was accessed 5 times at baseline, after artificial caries creation, and after fluoride exposure and a mean was calculated (value after the column that is colored). Samples were grouped based on mean values above (blue color) and below (Yellow color) the mean of the total sample.
(XLSX)
