# Supplementary material for: Aquaporin 5 Interacts with Fluoride and Possibly Protects against Caries
Source: PLoS One. 2015 Dec 2;10(12):e0143068. doi: 10.1371/journal.pone.0143068 (PMC4668048; doi:10.1371/journal.pone.0143068)
Supplement: S1 Table — (DOCX) [file pone.0143068.s001.docx]

Supplemental Material: Medications that cause Xerostomia:

http://www.drymouth.info/consumer/searchResults.asp

- Coumadin
- Topamax
- Lantus
- Lipitor
- Lactoluse
- Gabitril
- Digoxin
- Prilosec
- Aldactone
- Effexor
- Ortho evra
- Albuterol
- Protonix
- Elavil
- Synthroid
- Cardizem
- Nadolol
- Baclofen
- Zocor tabs
- Claritin-d
- Vicodin
- Lithium
- Amantadine
- Azmacort
- Perphenazine
- Imitrex
- Allegra-d
- Carbamazepine
- Diclofenac
- Metoprolol
- Warfarin
- Premarin
- Boniva
- Actonel
- Blood pressure medication
- Alprazolam
- Simvastatin
- Nexium
- Hydrochlorothiazide
- Ventolin
- Prilosec otc
- Hct
- Zyrtec
- Crixivan
- Norvasc
- Gabapentin
- Zyprexa
- Atropine
- Tramadol
- Luvox
- Singulair
- Excedrin
- Usinopril
- Inderal
- Lexapro
- Prozac
- Lamictal
- Kaletra
- Flomax
- Amlodipine
- Hydralazine
- Dyazide
- Ibuprofin
- Insulin
- Citalopram
- Lamictal
- Avapro
- Coreg
- Avalide
- Combivent
- Prevacid
- Zoloft
- Atenolol
- [st. John's wor](http://depression.emedtv.com/st.-john%27s-wort/st.-john%27s-wort-dosage.html)t
- Lamictal
- Lithobid
- Metoprolol
- Naproxen
- Gabapentin
- Truvada
- Neurontin
- Requip
- Lopressor
- Actonel
- Benicar
- Imitrex
- Morphine
- Paxil
- Cymbalta
- Lisinopril
- Amitriptyline
- Tegretol
- Detrol
- Lexapro
- Clozaril
- Nardil
- Dilt-cd
- Oxybutynin
- Diovan hct
- Seroquel
- Temazepam
- Flexeril
- Atropine
- Lyrica
- Wellbutrin
- Valium
- Ms contin
- Zanflex
- Celebrex
- Oxycontin
- Trazodone
- Dilaudid
- Seroquel
- Advair
- Gemfibrozil
- Micardis
- Famotidine
- Percocet
- Lexapro
- Buspar
- Risperdal
